# Identification of a novel dual-targeting peptide inhibitor of SARS-CoV-2 RBD and NRP1 through structure-based virtual screening

**DOI:** 10.3389/fphar.2025.1691679

**Published:** 2025-11-26

**Authors:** Nan Yao, Lixia Guan, Li Feng, Gang Wang, Yuting Wang, Miao-Miao Niu, Mei Yu, Shengnan Yin

**Affiliations:** 1 Department of Pharmacy, Taizhou Affiliated Hospital of Nanjing University of Chinese Medicine, Taizhou, China; 2 Department of Pharmaceutical Analysis, China Pharmaceutical University, Nanjing, China

**Keywords:** SARS-CoV-2, Omicron, RBD, NRP1, structure-based virtual screening

## Abstract

**Background:**

The continuous mutations of SARS-CoV-2 have enhanced its transmissibility and immune escape capabilities. Its invasion into cells relies on the binding of the receptor-binding domain (RBD) of the spike (S) protein to host receptors, while NRP1, as a key host cofactor, promotes viral attachment and internalization by recognizing the S protein. Therefore, dual targeting of RBD and NRP1 represents a potentially effective antiviral strategy.

**Methods:**

In this study, a multi-step screening strategy combining molecular docking, MST assays, molecular dynamics simulations, pseudovirus neutralization assays, MTT assays, and *in vivo* experiments in mice was used to discover dual-targeting inhibitors of SARS-CoV-2 RBD and NRP1.

**Results:**

Five peptide inhibitors (peptides 1–5) that simultaneously target RBD and NRP1 were identified through structure-based virtual screening. MST assays showed that peptides 1–5 all exhibited nanomolar affinity for both RBD and NRP1, with peptide-1 showing the strongest affinity for RBD (*K*_d_ = 19 nM) and NRP1 (*K*_d_ = 32 nM). Molecular dynamics simulations indicated that peptide-1 can stably bind to RBD and NRP1 proteins. Importantly, peptide-1 exerted efficient antiviral activity against the SARS-CoV-2 Omicron XBB.1.5 (EC_50_ = 0.62 ± 0.03 μM) and exhibited no obvious toxicity to normal human alveolar cells. Furthermore, *in vivo* assays indicated that peptide-1 had effective antiviral activity without severe side effects.

**Conclusion:**

In conclusion, peptide-1 is a highly effective and low-toxicity antiviral inhibitor that dual-targets RBD and NRP1.

## 1 Introduction

Since the outbreak of severe acute respiratory syndrome coronavirus 2 (SARS-CoV-2) in late 2019, coronavirus disease 2019 (COVID-19) caused by it has evolved into a major global public health crisis (Manirambona et al., 2024). The continuous mutation of the virus has been perpetually giving rise to variants with enhanced transmissibility and immune escape capability (Magazine et al., 2022). The S protein of the Omicron variant harbors multiple mutations, and the abundance of these mutations has raised significant concerns regarding the efficacy of current therapeutic vaccines and drugs (McCallum et al., 2022; Jawad et al., 2023). The Omicron variant has also spawned distinct subvariants (BA.1, BA.2, BA.3, BA.4, BA.5, etc.), among which BA.1, BA.2, and BA.5 have exhibited strong neutralization escape capabilities, posing significant challenges to vaccination and humoral immunity-based therapies (Uraki et al., 2022; Cao et al., 2023). As a significant clade within the Omicron lineage, the BA.2 subvariant harbors multiple critical mutations in its S protein, thereby exhibiting a high effective reproductive number, robust fusogenic capacity, and notable pathogenic potential (Yamasoba et al., 2022). Therefore, the Omicron BA.2 subvariant has precipitated sustained infection surges and imposed a substantial challenge to the therapeutic efficacy of currently available agents.

The invasion of host cells by SARS-CoV-2 is highly reliant on the interaction between its S protein and host receptors (Walls et al., 2020). The RBD of the S protein is responsible for the specific binding to the cellular receptor angiotensin-converting enzyme 2 (ACE2) and is subsequently cleaved by the host protease transmembrane serine protease 2 (TMPRSS2), which triggers the fusion of cellular and viral membranes, allowing viral RNA to enter the host cell and initiate the replication cycle (Walls et al., 2017; Lan et al., 2020; Hoffmann et al., 2020). Mutations in the RBD of the S protein can enhance its binding affinity to ACE2, which may consequently augment the viral transmissibility (Gobeil et al., 2021; Zahradník et al., 2021). The Omicron BA.2 subvariant harbors 16 mutations in its RBD, which significantly enhance its interaction with ACE2, thereby substantially increasing its infectivity (Chen and Wei, 2022). Studies have demonstrated that antibodies blocking the RBD-ACE2 interaction can directly mediate viral neutralization (Wang et al., 2020; Yu et al., 2020). Accordingly, targeting the RBD has emerged as a pivotal therapeutic strategy for combating viral infections.

Neuropilin-1 (NRP1), a key host factor involved in SARS-CoV-2 infection, can facilitate viral attachment and internalization by recognizing the C-terminal peptide motif of the S1 subunit of the S protein (Gudowska-Sawczuk and Mroczko, 2021; Chen et al., 2024). Studies have shown that blocking the interaction between NRP1 and the C-terminal fragment of the viral S1 subunit can reduce infection efficiency (Daly et al., 2020). Furthermore, in the context of co-expression of NRP1 and ACE2, TMPRSS2 can significantly promote the viral infection process (Cantuti-Castelvetri et al., 2020; Kyrou et al., 2021). Notably, NRP1 is highly expressed in respiratory and olfactory epithelial cells, and its expression is upregulated in the lung tissues of COVID-19 patients, which is closely associated with the severity of pulmonary symptoms (Cantuti-Castelvetri et al., 2020; Daly et al., 2020). In summary, NRP1 acts as an accessory factor in the process of viral entry, thus providing a new therapeutic target for antiviral interventions.

Currently, interventions targeting SARS-CoV-2 still have significant limitations. Vaccines show reduced protective efficacy due to the rapid mutation of the virus, while single-target inhibitors struggle to cope with the multi-pathway invasion mechanisms of the virus and are prone to inducing drug resistance due to their single target (Fischer and Feys, 2023). The high mutation rate of Omicron BA.2 increases its risk of escaping single-target drugs (Muruganantham and Veerabathiran, 2024), thus making the development of multi-target inhibitors capable of simultaneously blocking key steps in viral entry an urgent need. Given the conservation and synergistic roles of RBD and NRP1 in the entry of SARS-CoV-2 into host cells, the dual-targeting strategy against these two targets is expected to exert its effects through a dual-blockade mechanism: it not only inhibits the binding between RBD and ACE2, but also blocks the NRP1-mediated auxiliary entry, thereby effectively preventing viral internalization into host cells and reducing the risk of drug resistance induced by mutations in a single target. Peptide inhibitors exhibit distinct advantages in antiviral applications owing to their high specificity, low toxicity, and strong binding affinity for targets (Li et al., 2021; Nsereko et al., 2025). In this study, we focus on the identification of peptide inhibitors that dual target RBD and NRP1, with the aim of providing a novel therapeutic strategy to address infections caused by mutant strains.

Structure-based virtual screening is a low-cost and highly efficient drug discovery tool, which can be utilized to screen potential lead compounds against specific targets from large databases (Yin et al., 2022; Barril et al., 2004). Molecular docking screening can predict the binding conformations between ligands and targets to evaluate their binding affinity, thereby identifying drug candidates (Hosseini et al., 2021; Zhang et al., 2022). In previous studies, we have successfully identified a variety of novel inhibitors through structure-based virtual screening (Zhou et al., 2022; Yang et al., 2020; Zheng et al., 2021; Zheng et al., 2023; Zheng et al., 2024; Zhou et al., 2021). Here, we identified a novel, potent antiviral peptide inhibitor that dual-targets RBD and NRP1 through structure-based virtual screening. First, molecular docking screening of the database was performed based on the structure of RBD. Subsequently, the screened peptides were further docked to the active site of NRP1, and 5 potential peptides (peptides 1–5) were finally identified. These peptides 1–5 showed nanomolar-level binding affinities to both RBD and NRP1, with peptide-1 exhibiting the strongest binding affinity. Importantly, peptide-1 exerted efficient antiviral activity against the SARS-CoV-2 Omicron XBB.1.5 pseudovirus and showed no obvious toxicity to normal human alveolar cells. Moreover, peptide-1 exhibited effective antiviral activity in mice and caused no severe side effects. In conclusion, our study identified a dual-target peptide inhibitor of RBD and NRP1, which possesses high antiviral efficacy and low toxicity and is worthy of further investigation.

## 2 Materials and methods

### 2.1 Materials

The cells were purchased from the American Type Culture Collection (ATCC) (Manassas, VA, United States). PP-4, NTP, and peptides 1–5 were purchased from WuXi AppTec (Shanghai, China). The RBD and NRP1 proteins were purchased from Abcam (Cambridge, MA, United States).

### 2.2 Virtual screening

Molecular docking screening was performed using the Dock module of Molecular Operating Environment (MOE) software from a database containing 160,000 peptides. First, the three-dimensional crystal structures of RBD (PDB ID: 7DQA) and NRP1 (PDB ID: 7JJC) were obtained from the Protein Data Bank. Next, the QuickPrep module of MOE was used to optimize the structures of the two proteins, including removing water molecules, adding hydrogen atoms, and performing energy minimization. MOE’s Conformation Import tool converted the 2D chemical structures of molecules in the 160,000-peptide database into 3D structures. Molecular docking was conducted using the triangle matching algorithm of the Dock tool, and the binding free energy was calculated via the dG docking score. The lower the binding free energy, the stronger the binding affinity. All peptides were first docked to the active site of RBD, and the top 50 peptides with the lowest docking scores were selected; these were then docked to the active site of NRP1. Finally, the top 5 peptides with the lowest docking scores were selected for subsequent studies.

### 2.3 Microscale thermophoresis (MST) analysis

MST assay was used to analyze the binding ability of peptides 1–5 to RBD and NRP1. Specifically, RBD and NRP1 proteins were labeled using a lysine (Lys) labeling kit, respectively, and their final concentrations were adjusted to 50 nM. The peptides were serially diluted in a 1:1 gradient starting from an initial concentration of 12.5 μM. Subsequently, the labeled proteins were mixed with the diluted peptides in 100 mM potassium phosphate buffer (pH 7.0). After incubation at room temperature for 5 min, the mixture was loaded into standard MST glass capillaries. Detection was performed using a Monolith NT.115 instrument with the MST power set to 50% and the LED power set to 20%. The experiment was independently repeated three times.

### 2.4 Molecular dynamics (MD) simulations

MD simulations of protein-peptide complexes were performed using GROMACS to analyze their time-evolving stability characteristics in depth. The topological files for each complex system were accurately constructed under AMBER99SB-ILDN force field. During the simulation, each complex system was placed in a cubic solvent box with an edge length of 1.0 nm. Water molecules were described using the extended simple point charge (SPC/E) model, and charge neutralization was achieved by adding Na^+^ and Cl^−^. Subsequently, energy minimization of the system was carried out for 5,000 steps using the steepest descent method. The equilibration process was divided into two stages: first, the temperature was maintained at 300 K under the NVT ensemble using a V-rescale thermostat, and then the pressure was maintained at 1 atm under the NPT ensemble using a Parinello-Rahman pressure controller. Finally, 150 ns MD simulations were performed for each complex system.

### 2.5 Pseudovirus neutralization assay

Based on the HIV-1 pseudotyping technology, the RBD protein gene of the Omicron BA.2 variant was co-transfected into 293T cells together with a vector containing the firefly luciferase reporter gene to prepare pseudoviruses. 293T-RBD^+^/NRP1^+^ cells were seeded into 96-well plates at a density of 2 × 10^4^ cells/well and incubated overnight in a 37 °C, 5% CO_2_ incubator. There are two independent experiments: the first involves mixing 3.2 μM of peptides 1-5, PP-4, and NTP separately with 50 μL of pseudovirus at a 1:1 volume ratio, followed by incubation at room temperature for 1 h; the second involves mixing serially diluted peptide-1 (at concentrations of 0, 0.2, 0.4, 0.8, 1.6, 3.2, 6.4, and 12.8 μM) separately with 50 μL of pseudovirus at a 1:1 volume ratio, and incubating at room temperature for 1 h. After the reactions in both experiments were completed, the old medium in the 96-well plates was aspirated, and 100 μL of the corresponding virus-peptide mixture was added to each well. The plates were then incubated in a 37 °C, 5% CO_2_ incubator for 48 h to allow the virus to complete its infection cycle. At 48 h post-infection, luciferase activity was measured according to the instructions of the luciferase detection kit. The formula for calculating the infection rate (%) is: (Luminescence signal of test sample/Luminescence signal of negative control sample) × 100.

### 2.6 hERG fluorescence polarization assay

This study was conducted using the Predictor hERG Fluorescence Polarization Assay Kit purchased from Invitrogen. The kit comprises Predictor hERG Tracer Red, Predictor hERG Membrane, Predictor hERG FP Assay Buffer, and E-4031 as the positive control. The Predictor hERG Tracer Red was diluted to prepare a 4 nM tracer working solution, while the stock solution of E-4031 was diluted to a 120 μM E-4031 working solution. Subsequently, serial dilutions of E-4031 were prepared to serve as the reference compound. Test peptides were subjected to gradient dilution to generate solutions with 6 concentration gradients. For the assay procedure, buffer or compound solution was first added to the assay plate, followed by the addition of membrane and the tracer. After incubating the assay plate at 25 °C for 4 h, fluorescence polarization (FP) values (mp values) were measured using an EnVision microplate reader (PerkinElmer) equipped with a set of polarized filters. The half-maximal inhibitory concentration (IC_50_) of active compounds was determined using GraphPad Prism software.

### 2.7 MTT assay

A549 cells (NHBE, catalog number: PCS-300-010) were purchased from the American Type Culture Collection (ATCC, Manassas, VA, United States). The cells were cultured in Airway Epithelial Cell Basal Medium (ATCC, catalog number: PCS-300-030) supplemented with Bronchial/Tracheal Epithelial Cell Growth Kit (ATCC, catalog number: PCS-300-040) and incubated in a 37 °C incubator with 5% CO_2_. Then, cells were seeded into 96-well plates at a density of 5 × 10^4^ cells per well and incubated overnight at 37 °C with 5% CO_2_. Subsequently, cells were treated with 100 μM peptides 1–5 for 48 h. After treatment, MTT stock solution (0.5 mg/mL) was added to each well, followed by additional 4-h incubation. Finally, the absorbance signal was measured at 570 nm using a microplate reader.

### 2.8 *In vivo* efficacy studies

All experiments were conducted in the biosafety level 3 (BSL3) laboratory and animal biosafety level 3 (ABSL3) facility of the Wuhan Institute of Virology, Chinese Academy of Sciences (WIV, CAS). These facilities are certified by the National Health Commission of the People’s Republic of China and comply with the national standards GB 19489-2018 (General Requirements for Laboratory Biosafety) and GB 50447-2019 (Technical Specifications for Construction of Animal Biosafety Laboratories), equipped with negative pressure ventilation systems, dedicated biosafety cabinets, and full sets of personal protective equipment to ensure safe handling of live SARS-CoV-2 and infected animals.

All animal experimental protocols were approved by the Animal Experiments Committee of Wuhan Institute of Virology, Chinese Academy of Sciences [WIVA17202005]. The experiments strictly followed the “Guide for the Care and Use of Laboratory Animals” (8th edition, National Academies Press, 2011) and adhered to the 3R principles (Replacement, Reduction, Refinement). Efforts were made to minimize the number of animals while ensuring statistical power, and humane endpoints were applied throughout the study—euthanasia was performed via carbon dioxide inhalation following standardized procedures to reduce animal suffering.

Female K18-hACE2 mice (6–8 weeks old) were purchased from GemPharmatech Co., Ltd. (Nanjing, China) and acclimated to the ABSL3 facility environment for 3 days prior to experimentation. The mice were randomly divided into three groups (n = 5) using a random number table: two experimental groups intranasally administered peptide-1 at doses of 1 mg/kg and 5 mg/kg, respectively, and a control group receiving an equal volume of PBS. At 2 h post-administration, all mice were inoculated intranasally with 10,000 50% tissue culture infective dose (TCID50) of live SARS-CoV-2 Omicron XBB.1.5 strain.

On day 2 post-virus inoculation, mice were euthanized, and lung tissues were rapidly harvested under sterile conditions. After rinsing with pre-cooled PBS to remove blood contaminants, tissue homogenates were prepared at a ratio of 1 mL PBS per 100 mg of lung tissue using a tissue homogenizer. The homogenates were centrifuged at 12,000 rpm for 10 min at 4 °C, and the supernatants were collected for virus load detection via the focus reduction neutralization test (FRNT). Additionally, the levels of liver and renal function-related parameters were measured using a biochemistry automatic analyzer (Cobas Integra 400 Roche, Germany). All experimental operations strictly complied with the biosafety regulations of WIV, CAS and national biosafety management requirements for pathogenic microorganisms.

## 3 Results

### 3.1 Virtual screening

**FIGURE 1:**
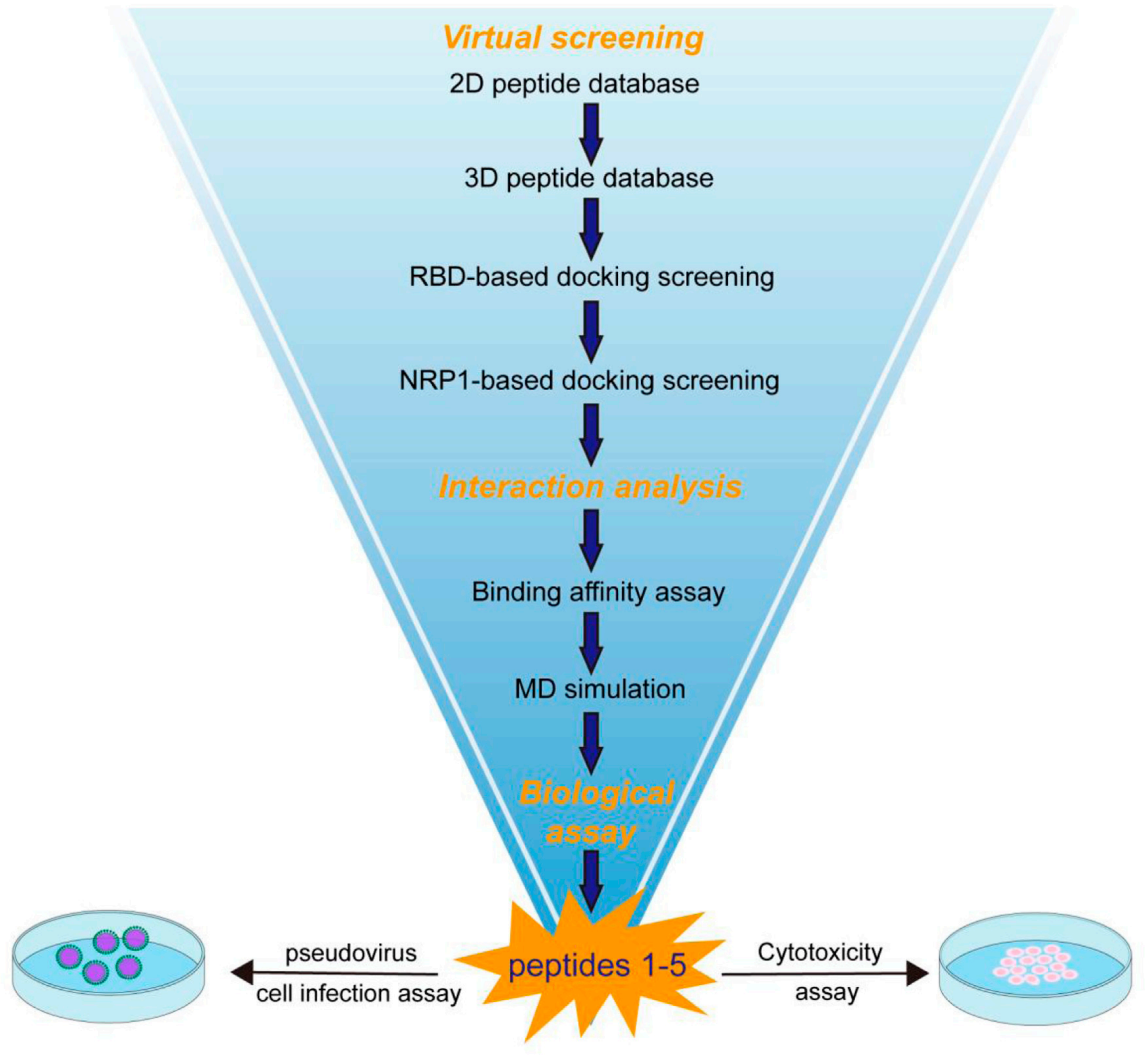
The multi-stage virtual screening workflow.

To identify highly effective and potential dual-target inhibitors against RBD and NRP1, this study established a multi-stage virtual screening workflow as shown in Figure 1. First, the 2D peptide library containing 160,000 peptides was converted into stable 3D conformations. Subsequently, using the crystal structure of SARS-CoV-2 RBD (PDB ID: 7DQA) as the receptor, rigid-receptor semi-flexible docking was performed via the MOE-Dock module. The lower the binding free energy, the higher the binding affinity. With the docking binding energy as the evaluation index, the top 50 RBD-targeting peptides with the lowest binding free energy were retained. These 50 candidate peptides were then further docked to the NRP1 structure (PDB ID: 7JJC) with consistent docking parameters. Finally, the top 5 peptides (designated as peptides 1–5) that exhibited the lowest binding free energy were selected. These peptides 1–5 target both RBD and NRP1 simultaneously and were used for subsequent *in vitro* activity validation.

### 3.2 Interaction analysis

**FIGURE 2:**
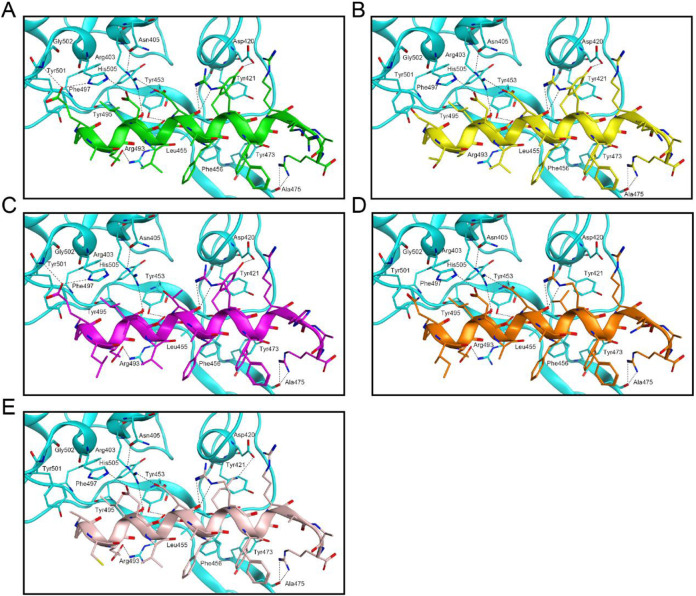
Interaction analyses of peptides 1–5 with RBD. **(A)** Conformation of peptide-1 at the RBD binding site; **(B)** Conformation of peptide-2 at the RBD binding site; **(C)** Conformation of peptide-3 at the RBD binding site; **(D)** Conformation of peptide-4 at the RBD binding site; **(E)** Conformation of peptide-5 at the RBD binding site. Residues of RBD at the active site are shown in cyan. Hydrogen bonds are indicated by black dashed lines.

**FIGURE 3:**
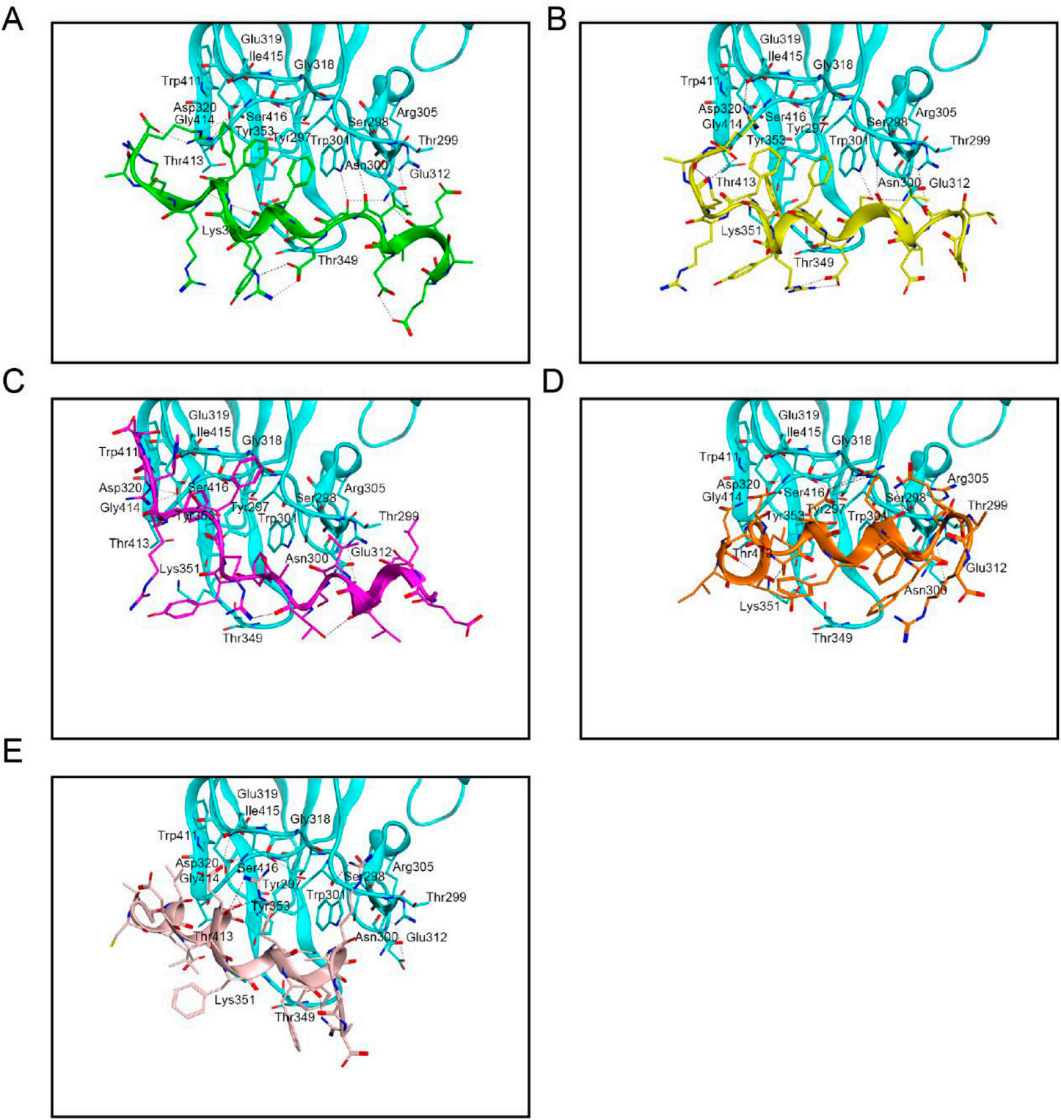
Interaction analyses of peptides 1-5 with NRP1. **(A)** Conformation of peptide-1 at the NRP1 binding site; **(B)** Conformation of peptide-2 at the NRP1 binding site; **(C)** Conformation of peptide-3 at the NRP1 binding site; **(D)** Conformation of peptide-4 at the NRP1 binding site; **(E)** Conformation of peptide-5 at the NRP1 binding site. Residues of NRP1 at the active site are shown in cyan. Hydrogen bonds are indicated by black dashed lines.

Interaction analyses were performed to clarify the binding modes of peptides 1–5 with RBD and NRP1, respectively. Figure 2 shows the binding model analysis between peptides 1–5 and the RBD protein. The key amino acids at the active site of the RBD protein, including Arg403, Asp420, Tyr453, Ala475, Arg493, Gly502, and His505, formed hydrogen bond interactions with peptides 1–5. Among them, peptide-1 established 10 hydrogen bonds with these amino acids; peptide-2 and peptide-4 each formed 7 hydrogen bonds with some of these amino acids; and peptide-3 and peptide-5 each formed 8 hydrogen bonds with some of these amino acids. Meanwhile, peptides 1–5 created hydrophobic interactions with the amino acids at the active site of the RBD protein, such as Tyr421, Leu455, Phe456, Tyr473, Tyr495, Phe497, and Tyr501. Figure 3 presents the interaction analysis between peptides 1–5 and the NRP1 protein. The peptide-1 formed 9 hydrogen bonds with the key amino acids Ser298, Asn300, Trp301, Asp320, Thr413, and Lys351 of the NRP1 protein. The peptide-2 formed 6 hydrogen bonds with the key amino acids of the NRP1 protein, including Ser298, Asn300, Trp301, Glu319, Asp320, and Lys351. The peptides 3–5 each formed 3 hydrogen bonds with key amino acids of the NRP1 protein, with peptide-3 interacting with Asn300 and Asp320; peptide-4 with Asp320 and Lys351; and peptide-5 with Tyr297 and Asp320. In addition, peptides 1–5 exhibited key hydrophobic interactions with the amino acids Tyr297, Gly318, Tyr353, Gly414, and Ile415 at the active site of the NRP1 protein. In general, peptides 1–5 bind to RBD and NRP1 through hydrogen bond interactions and hydrophobic interactions. Among them, peptide-1 may exhibit stronger binding capacity due to more hydrogen-bond interactions with RBD and NRP1.

### 3.3 Binding affinity assay

The binding capacity of peptides 1–5 with RBD and NRP1 was evaluated by MST assay. As shown in Table 1, peptides 1–5 exhibited nanomolar affinity for both RBD and NRP1, with dissociation constant (*K*_d_ values) ranges of 19–69 nM and 32–96 nM, respectively. Among them, peptide-1 had the most potent binding affinity targeting RBD (*K*_d_ = 19 nM) and NRP1 (*K*_d_ = 32 nM). In addition, we evaluated the binding affinities of PP-4 (a positive RBD-targeting inhibitor) and NTP (a positive NRP1-targeting inhibitor) with RBD and NRP1 proteins, respectively. The results showed that PP-4 exhibited strong binding affinity for RBD (*K*_d_ = 79 nM) but no binding affinity for NRP1; NTP exhibited strong binding affinity for NRP1 (*K*_d_ = 141 nM) but no binding affinity for RBD (Table 1). Compared with the single-target peptides, peptide-1 showed approximately 4.2-fold and 4.4-fold higher affinity for RBD and NRP1, respectively, suggesting its potential for efficient dual-target intervention.

**TABLE 1:** Binding affinities of peptides 1–5 to RBD and NRP1.

Name	Sequence	RBD (*K*_d_, nM)	NRP1 (*K*_d_, nM)
Peptide-1	EAELdVSDAFYRFFRRAR	19	32
Peptide-2	STALdVSDAFYRFFRKAR	56	48
Peptide-3	ELELVVSTAFYRFFRHAR	64	81
Peptide-4	NIELdASDAFVRFFRQAR	69	75
Peptide-5	ACEIdVSDAFKRFFRAAR	43	96
PP-4	LVMGLNVWLRYSK-βA-K(Biotin)-CONH_2_	79	No binding
NTP	rppregr	No binding	141

### 3.4 MD simulations

**FIGURE 4:**
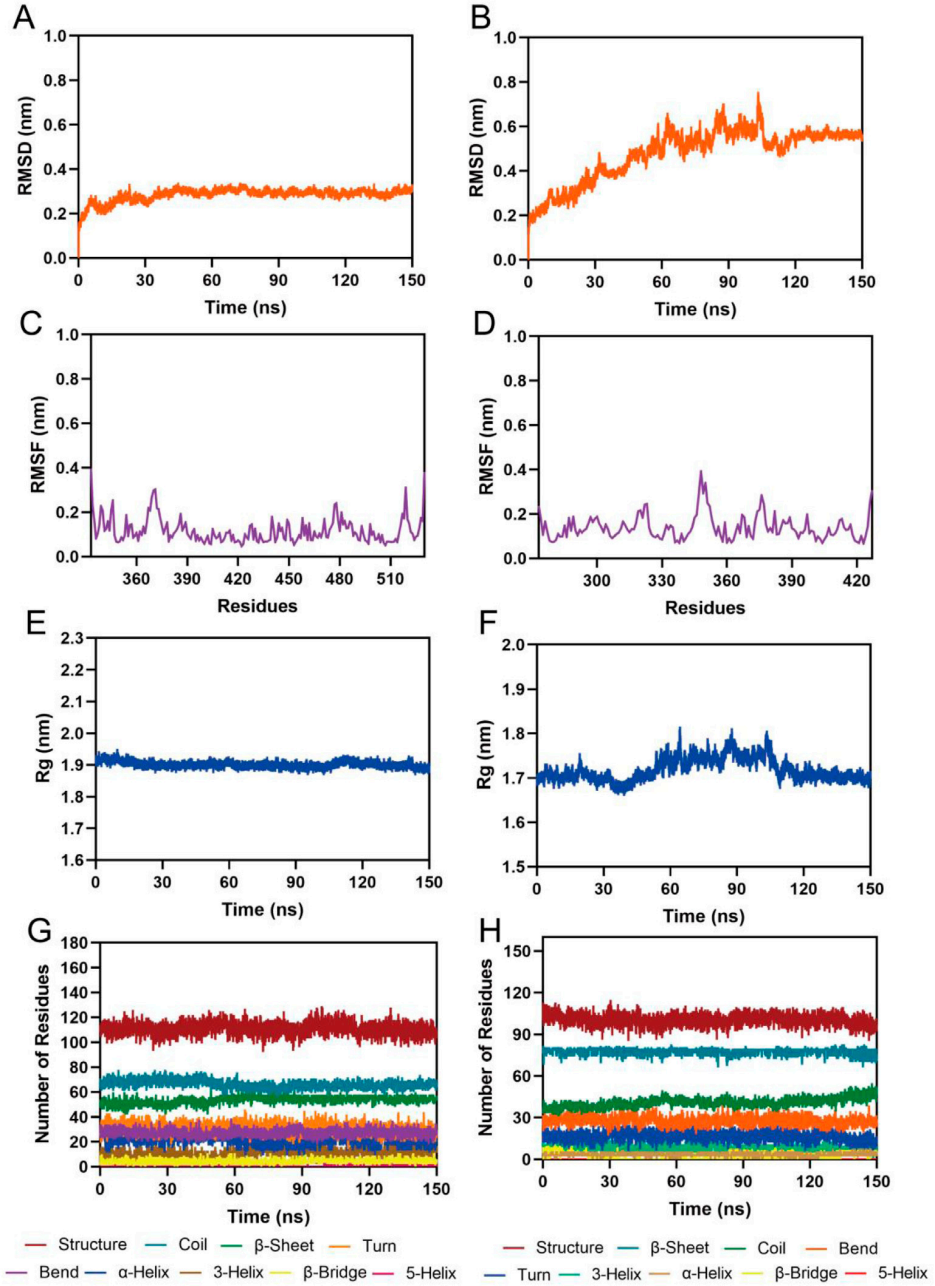
MD simulation of peptide-1 with RBD and NRP1. **(A,B)** RMSD values of the peptide-1-RBD complex and peptide-1-NRP1 complex, respectively. **(C,D)** RMSF values of RBD and NRP1 residues in the peptide-1-RBD complex and peptide-1-NRP1 complex, respectively. **(E,F)** Rg values of RBD and NRP1 in the peptide-1-RBD complex and peptide-1-NRP1 complex, respectively. **(G,H)** Secondary structures of RBD and NRP1 in the peptide-1-RBD complex and peptide-1-NRP1 complex, respectively.

To evaluate the binding stability of the peptide-1-RBD and peptide-1-NRP1 complexes, we performed 150 ns MD simulations using GROMACS. The stability of the protein-ligand complexes was analyzed through key MD parameters: root mean square deviation (RMSD), root mean square fluctuation (RMSF), radius of gyration (Rg), and protein secondary structure. RMSD is a critical indicator of system convergence. As shown in Figure 4A, the RMSD value of the peptide-1-RBD complex increased slightly initially and then stabilized at approximately 0.28 nm, suggesting that peptide-1 forms a stable complex with RBD. The RMSD of the peptide-1-NRP1 complex initially increased gradually, which was caused by the complex adapting to the simulation environment and adjusting its structural conformation from the initial state. After approximately 60 ns, the RMSD stabilized and fluctuated slightly around 0.55 nm, indicating that the complex reached a relatively stable structural state (Figure 4B). RMSF is used to evaluate the flexibility of key residues. As depicted in Figures 4C,D, the RMSF values of key residues (Arg403, Asp420, Tyr453, Ala475, Arg493, Gly502, His505, Tyr421, Leu455, Phe456, Tyr473, Tyr495, Phe497, and Tyr501) in the RBD binding site were all below 0.15 nm. The RMSF values of key residues (Ser298, Asn300, Trp301, Asp320, Thr413, Lys351, Glu319, Tyr297, Gly318, Tyr353, Gly414, and Ile415) in the NRP1 binding site were all below 0.25 nm. This suggests that these key residues form stable interactions with peptide-1. The Rg values of RBD and NRP1, which measure protein compactness, fluctuated around 1.9 nm and 1.7 nm, respectively (Figures 4E,F). This indicates that the binding of peptide-1 maintains the structural integrity of RBD and NRP1. Secondary structure analysis further confirmed that the binding of peptide-1 did not cause significant changes in the conformational structures of RBD and NRP1 (Figures 4G,H). In summary, these results demonstrate that peptide-1 can bind stably to RBD and NRP1 proteins, maintaining the structural stability of the complexes during the simulation.

### 3.5 Inhibitory effect on pseudotyped SARS-CoV-2 infection

**FIGURE 5:**
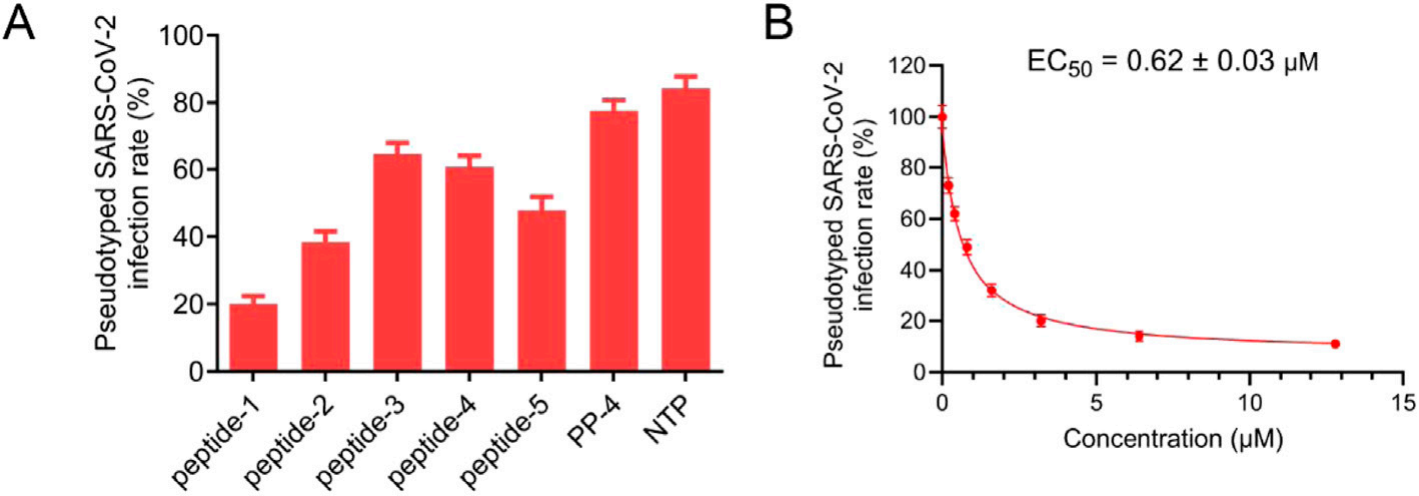
Pseudovirus infection inhibitory activities of peptides 1-5. **(A)** Infection rates of pseudotyped SARS-CoV-2 Omicron XBB.1.5 treated with peptides 1-5, PP-4, and NTP at a concentration of 3.2 μM. **(B)** Dose-dependent assay of peptide-1 (at concentrations of 0, 0.2, 0.4, 0.8, 1.6, 3.2, 6.4, and 12.8 μM) against SARS-CoV-2 variant Omicron XBB.1.5. Results are presented as mean ± standard deviation, n = 3.

Owing to the high infectivity and pathogenicity of SARS-CoV-2, research involving this virus presents inherent challenges and risks. The pseudovirus system, characterized by high safety, a broad host range, and genetic stability, serves as a safe and effective alternative tool for SARS-CoV-2 entry experiments. Additionally, previous studies have reported good consistency between pseudovirus neutralization assays and SARS-CoV-2 neutralization assays. Therefore, to evaluate the inhibitory effects of peptides 1–5, we employed the SARS-CoV-2 pseudovirus infection assay for preliminary assessment. As shown in Figure 5A, at a concentration of 3.2 μM, the inhibitory activities of peptides 1–5 against the SARS-CoV-2 Omicron XBB.1.5 pseudovirus were all stronger than those of the positive controls PP-4 and NTP. Among them, peptide-1 exhibited the most significant inhibitory effect, with an inhibition rate of approximately 80% against SARS-CoV-2 Omicron XBB.1.5 infection. Further experiments demonstrated that the half-maximal effective concentration (EC_50_) of peptide-1 against the SARS-CoV-2 pseudovirus variant Omicron XBB.1.5 was 0.62 ± 0.03 μM (Figure 5B). These results indicate that peptide-1 possesses efficient antiviral activity.

### 3.6 Toxicity prediction of peptides 1–5

A predictive analysis of the toxicity profile of peptides 1–5 was conducted using the ADMETlab platform (https://admet.scbdd.com/). As shown in Table 2, peptides 1–5 exhibited minimal Ames mutagenicity and human hepatotoxicity, with an extremely low probability of inducing the aforementioned toxicities. This indicates that peptides 1–5 possess favorable safety profiles in terms of potential carcinogenicity and hepatotoxicity. However, the predictive results revealed that peptides 1–5 exerted a moderate degree of blocking activity against the hERG potassium ion channel, and this finding suggests that peptides 1–5 may harbor latent cardiotoxicity risks.

**TABLE 2:** Toxicity prediction results of peptides 1-5.

Name	AMES (Ames mutagenicity)		hERG (hERG blockers)		H-HT (human hepatotoxicity)	
	Category^a^	Probability^b^	Category^a^	Probability^b^	Category^a^	Probability^b^
Peptide-1	0	0.116	1	0.602	0	0
Peptide-2	0	0.116	1	0.602	0	0
Peptide-3	0	0.13	1	0.62	0	0
Peptide-4	0	0.128	1	0.574	0	0
Peptide-5	0	0.122	1	0.582	0	0

^a^ Category 0: negative (−); Category 1: positive (+).

^b^ The Probability for the Category means probability of predicted category 1.

### 3.7 hERG toxicity assays

Considering the aforementioned toxicity prediction results suggesting that peptides 1–5 may pose cardiotoxicity risks, we further conducted hERG toxicity assays. As shown in Table 3, the IC_50_ of peptides 1–5 against the hERG channel all exceeded 50 μM. This result indicates that within the tested concentration range, peptides 1–5 exhibited no significant inhibitory activity toward the hERG channel, thereby demonstrating favorable cardiac electrophysiological safety profiles and a low risk of cardiotoxicity.

**TABLE 3:** hERG toxicity results of peptides 1–5.

Name	hERG IC_50_ (μM)
Peptide-1	>50
Peptide-2	>50
Peptide-3	>50
Peptide-4	>50
Peptide-5	>50

### 3.8 Safety profiles of peptides 1–5

**FIGURE 6:**
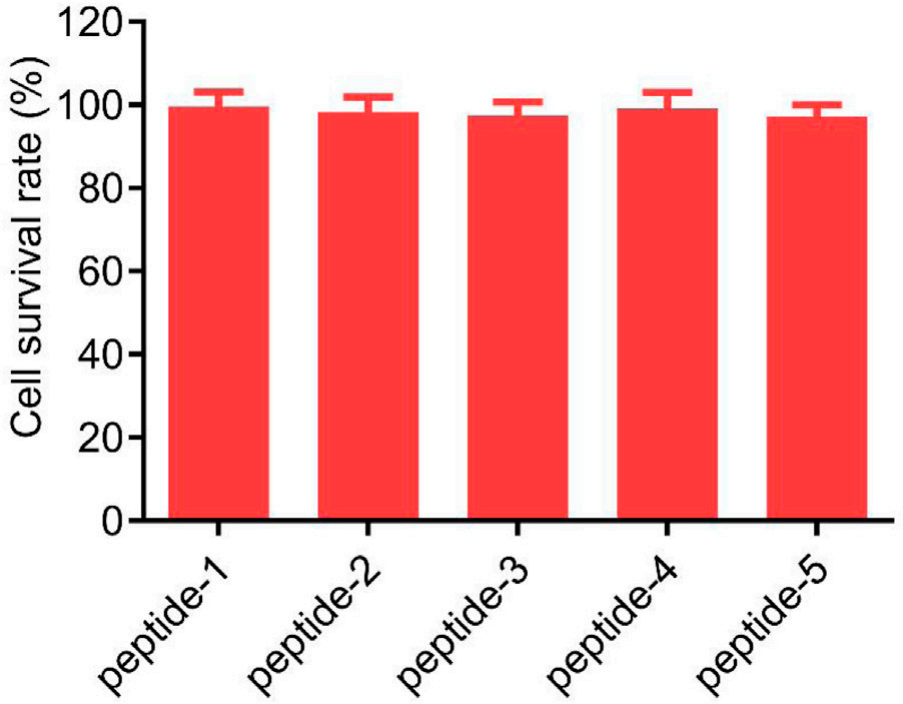
Cytotoxicity of peptides 1–5 on A549 cells detected by MTT assay. Cells were treated with 100 μM peptides 1–5 for 48 h. Results are presented as mean ± standard deviation, n = 3.

Patients with COVID-19 may develop pulmonary symptoms, and SARS-CoV-2 has been detected in the lungs. Therefore, human alveolar basal epithelial cells (A549), which are relevant to the pulmonary manifestations of COVID-19 patients, were used to further assess the toxicity of peptides 1–5. After treating the cells with a high concentration of 100 μM for 48 h, the results showed that peptides 1–5 did not exhibit observable cytotoxicity to A549 cells (Figure 6), indicating that peptides 1–5 have excellent safety.

### 3.9 *In vivo* efficacy studies

**FIGURE 7:**
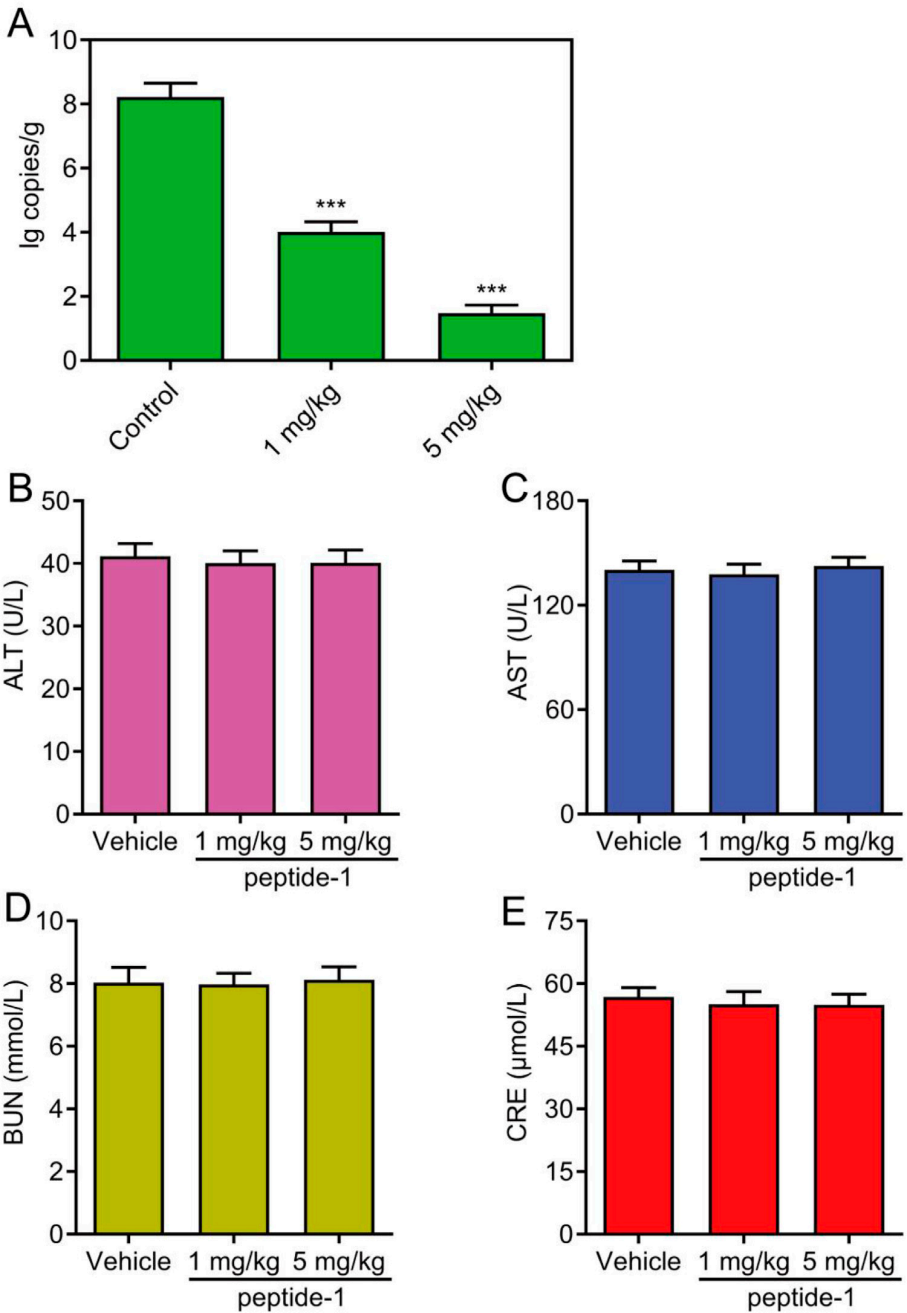
Evaluation of the antiviral activity of peptide-1 in mice. **(A)** Peptide-1 significantly reduced the pulmonary viral load in mice after infection with the Omicron XBB.1.5 strain. **(B–E)** Biochemical parameters were measured in each group of mice. Data are presented as mean ± SD, n = 5, ***p < 0.001.

The antiviral efficacy of peptide-1 in mice was further evaluated. As shown in Figure 7A, compared with the control group, peptide-1 at doses of 1 mg/kg and 5 mg/kg both significantly reduced the viral load in the lungs of mice. Meanwhile, the viral load in the 5 mg/kg treatment group was lower than that in the 1 mg/kg treatment group, indicating that peptide-1 had a clear antiviral effect with a dose-dependent relationship. In addition, the potential toxic and side effects of peptide-1 were further assessed by monitoring the biochemical parameters in mice. The results showed that the levels of alanine aminotransferase (ALT), aspartate aminotransferase (AST), blood urea nitrogen (BUN), and creatinine (CRE) did not change significantly, confirming that the liver and kidney functions of mice were metabolically normal (Figures 7B–E). These results indicated that peptide-1 had effective antiviral activity without severe side effects.

## 4 Discussion

Since the emergence of SARS-CoV-2, continuous viral mutations have led to progressive increases in the transmissibility and immune escape capabilities of variants. The specific interaction between the RBD of the S protein and host receptors constitutes a pivotal step in viral entry into host cells. Concomitantly, NRP1, serving as a key host cofactor, facilitates viral attachment and internalization through its recognition of the S protein, thereby potentiating the infective process. Given the synergistic roles of RBD and NRP1 in the invasion process of SARS-CoV-2, dual targeting of these two proteins is anticipated to represent an effective antiviral strategy. In this study, peptide inhibitors (peptides 1–5) that simultaneously target RBD and NRP1 were identified through structure-based virtual screening. Peptides 1–5 bound to RBD and NRP1 via hydrogen bonding interactions and hydrophobic interactions. Among them, peptide-1 exhibited stronger binding due to more hydrogen bonding interactions with RBD and NRP1. MST experiments demonstrated that peptides 1–5 all possessed high nanomolar affinity for both RBD and NRP1, with peptide-1 showing the strongest affinity. MD simulations revealed that peptide-1 could stably bind to RBD and NRP1 proteins, and the structures of the complexes remained stable during the simulation. Importantly, peptide-1 exerted efficient antiviral activity against the SARS-CoV-2 Omicron XBB.1.5 pseudovirus and exhibited no obvious toxicity to normal human alveolar cells. Additionally, *in vivo* experiments showed that peptide-1 exhibited potent antiviral activity and caused no severe side effects. In summary, peptide-1 is a highly effective and low-toxicity antiviral inhibitor that dual-targets RBD and NRP1, providing a potential candidate molecule for the development of novel antiviral drugs against SARS-CoV-2.

## Data Availability

The original contributions presented in the study are included in the article/supplementary material, further inquiries can be directed to the corresponding authors.
